# Unravelling the Longitudinal Relations Between Developmental Milestones, General Psychopathology, and Personality Functioning in a Youth Clinical Sample

**DOI:** 10.1007/s10964-024-01971-2

**Published:** 2024-03-18

**Authors:** Sara Iannattone, Hilde D. Schuiringa, Anouk Aleva, Nagila Koster, Marcel A. G. van Aken, Christel J. Hessels, Paul T. van der Heijden, Odilia M. Laceulle

**Affiliations:** 1 Department of General Psychology, University of Padova, Padova, Italy; 2 Department of Developmental Psychology, Utrecht University, Utrecht, The Netherlands; 3 HYPE Centre of Expertise on Early Intervention for Borderline Personality Disorder, GGz Centraal, Amersfoort, The Netherlands; 4 Reinier van Arkel Mental Health Institute, ’s-Hertogenbosch, Noord-Brabant, The Netherlands; 5 Behavioural Science Institute, Radboud University Nijmegen, Nijmegen, The Netherlands

**Keywords:** Adolescence, Cross-lagged panel model, Emerging adulthood, Longitudinal study, Patients

## Abstract

Personality functioning, general psychopathology, and developmental milestones achievement are critical domains in the field of young people’s mental health; however, no prior research has considered these variables jointly or examined the temporal dynamics between them. To fill these gaps, the present study aimed to investigate the longitudinal associations between the above constructs in a clinical sample of Dutch youth. 525 outpatients (72.5% women; age range: 12–26 years, *M* = 18.8 ± 2.83) diagnosed with different psychological difficulties were recruited from specialized mental health care services in The Netherlands. They completed self-report measures assessing personality functioning, psychopathological symptoms, and the achievement of youth-specific developmental milestones. Data were collected on three occasions within a year and modelled using a Cross-Lagged Panel Model approach. The levels of personality dysfunction, general psychopathology, and developmental milestones achievement were found to fluctuate from one wave to the other. Personality dysfunction and general psychopathology were positively interrelated at each time point, while both constructs were negatively associated with developmental milestones achievement. Importantly, difficulties achieving developmental milestones predicted a worsening in personality functioning 6 months later. This result would suggest that the achievement of developmental milestones precedes personality functioning, supporting the importance of interventions promoting age-adequate functioning in youth.

## Introduction

Adolescents and emerging adults frequently struggle with mental health problems, which often lead to enduring maladaptive consequences (Blakemore, 2019). In fact, most mental disorders emerge between the ages of 11 and 21 years (Kessler et al., 2007; Solmi et al., 2022), with internalizing problems typically more prevalent in girls and externalizing problems in boys (Muratori et al., 2021). Therefore, investigating the mechanisms underlying youth psychopathology is crucial for both treatment and prevention purposes. Impairment in personality functioning is a relevant construct within this context, as it has been shown to cut across a wide range of psychopathological symptoms (e.g., Sleep et al., 2019). Furthermore, the field has advocated for the importance of adopting a developmental perspective to (the emergence of) psychopathology in youth (De Fruyt & De Clercq, 2014; Thapar & Riglin, 2020); to this end, the achievement of youth-specific developmental milestones should be considered (Sharp, 2020). Indeed, adolescence and emerging adulthood is a critical period for achieving salient developmental milestones, primarily regarding peer relationships (social domain), identity formation and autonomy (personal domain), and academic/working performance (professional domain) (Feldman et al., 1990). These milestones are unique yet interdependent, thus converging into the unified concept of “milestone achievement” (Seiffge-Krenke & Gelhaar, 2008). Extensive research has supported the interplay between developmental milestones achievement and general psychopathology in youth (e.g., Allen et al., 2022; Potterton et al., 2022); however, the direction of this association remains unclear. Moreover, while there is theoretical support for the relation between developmental tasks achievement and personality functioning (Sharp, 2020), empirical evidence is currently lacking. Therefore, this longitudinal study aimed to bridge existing gaps by examining the interplay between personality functioning, general psychopathology, and developmental milestones achievement in an outpatient sample of youth.

### The Relation Between Personality Functioning and General Psychopathology

Personality Disorders (PDs) frequently have their onset in adolescence and emerging adulthood (Chanen & Thompson, 2019; Sharp & De Clercq, 2020). Literature has indicated heterogeneity in pathways to PDs. For example, some studies suggested that PD exacerbation would be preceded and nurtured by internalizing and externalizing problems (Benzi et al., 2023; Stepp et al., 2016), which would then remain comorbid with PDs throughout development (Sharp & Wall, 2018). At the same, it has also been shown that maladaptive personality traits are already present in childhood, posing a risk for later development of internalizing and externalizing problems (De Clercq et al., 2006, 2009). Currently, PDs are conceptualized and assessed dimensionally (Hopwood et al., 2018), for example through the lens of the DSM-5 Alternative Model of Personality Disorders (AMPD; American Psychiatric Association, 2013). Unlike the traditional categorical symptom-based approach, a dimensional approach is deemed more developmentally sensitive (Sharp, 2020) and therewith more useful in the conceptualization of personality pathology in youth (Sharp et al., 2018; Weekers et al., 2021). In the view of the AMPD, PDs are characterized not only by the presence of maladaptive personality traits (Criterion B), but also by impairments in the global level of personality functioning (Criterion A). The latter includes two components: self- and interpersonal functioning; specifically, self-functioning is composed of identity and self-direction domains, while interpersonal functioning comprises empathy and intimacy domains.

To date, personality functioning has been studied mainly in relation to PDs; nevertheless, some degree of impairment in personality functioning can also occur in other psychopathologies (Bach, 2018). For example, associations between impairments in personality functioning and different mental disorders, such as posttraumatic stress disorder (Møller et al., 2021), eating disorders (Klein et al., 2022), depression (Sleep et al., 2019; Vittengl et al., 2023), and anxiety disorders (Doering et al., 2018; Gruber et al., 2020), have been found in adult samples. Furthermore, it has been suggested that impaired personality functioning may be an overall indicator of the presence and severity of a broad array of mental health problems (Doubková et al., 2022; Hengartner et al., 2014). Taken together, these results would seem to hint at the existence of an association between personality functioning and psychopathology.

Nonetheless, although research in this field is flourishing, much remains still unknown about (impairments in) personality functioning in other-than-PD mental disorders; in particular – and importantly, given the emergence of psychopathology – limited studies have investigated the relation between personality functioning and general psychopathology in youth samples. To best address this topic, a developmental perspective on psychopathology should be adopted (Holmbeck et al., 2006; Cicchetti & Rogosch, 2002). A primary developmentally-oriented variable to consider is the achievement of developmental milestones, which can be seen as a ‘benchmark’ of age-adequate functioning and thus is fundamental to evaluate in clinical research and practice (Holmbeck et al., 2006). Gender differences in the attainment of normative developmental tasks have been observed, albeit with limited available evidence. Particularly, 14–16-year-old female adolescents appear to show a greater overall level of developmental progression than their male peers, possibly due to the earlier maturation of adolescent girls (Seiffge-Krenke & Gelhaar, 2008). In general, the achievement of youth-specific developmental milestones is an important prerequisite for solving tasks in adulthood (e.g., Roisman et al., 2004), as well as a critical factor to long-term psychological well-being (e.g., Gómez-López et al., 2019); along the same line, failure to complete age-typical developmental tasks can lead to negative mental health outcomes (Pinquart & Pfeiffer, 2020). In support of the pivotal role played by the successful mastery of developmental milestones in the psychological adjustment of youth, a link (theoretical or empirical) has been shown between milestones and both general psychopathology and personality functioning.

### Youth-Specific Developmental Milestones and General Psychopathology

A sizeable body of research has explored the relation between developmental milestones and psychopathology in youth, despite providing contrasting results with respect to the direction of this association. For instance, pertaining to the social area, some longitudinal studies on community samples have shown that high psychopathology levels were predictive of peer conflict (H. Chen et al., 2009) and negative social interaction (Achterhof et al., 2022); conversely, other studies found that high-quality peer relationships can have a protective role against the subsequent onset of mental disorders in both adolescents and emerging adults (Allen et al., 2022; Kent & Bradshaw, 2021). In a similar vein, shifting the focus to the personal area, the direction of the link between identity and psychopathology in youth is unclear. Indeed, difficulties in identity formation are currently considered a transdiagnostic risk factor for a wide spectrum of mental disorders (Kaufman et al., 2014; Klimstra & Denissen, 2017); nevertheless, a negative influence of early psychopathology on identity development has also been found (Potterton et al., 2022). Finally, pertaining to the professional area, there is a scarcity of longitudinal evidence on the link between school/working performance and psychopathology, especially in emerging adults; however, cross-sectional studies on both clinical (Ogilvie et al., 2019) and non-clinical (Gonzálvez et al., 2022; Ligier et al., 2020) adolescent samples supported a reciprocal influence between school difficulties (e.g., academic failure, school avoidance and dropout, low investment in schoolwork) and symptoms of mental disorders.

Generally speaking, the models explaining the relation between developmental tasks and psychopathology can be grouped into four broad categories, as summarized by Masten et al. (2015): (1) Common Cause Models; (2) Psychopathology undermines competence; (3) Failure Models; and (4) Complex Dynamic Models. According to the Common Cause Models, difficulties achieving developmental tasks and psychopathology may stem from the same underlying processes manifesting in different ways (e.g., emotion regulation difficulties contribute to both impaired achievement of developmental milestones and various mental disorders). The subsequent category of models assumes that psychopathological symptoms may interfere with adaptive mastery of developmental tasks; on the contrary, in the view of the Failure Models, problems in accomplishing developmental tasks may foster greater psychopathological vulnerability. Finally, the Complex Dynamic Models posit that causal effects are often reciprocal or bidirectional; as a consequence, developmental tasks and psychopathology may be connected by complex mechanisms over time.

### Youth-Specific Developmental Milestones and Personality Functioning

In contrast to abundant research on developmental milestones and psychopathology, there is considerably less evidence regarding the relation between developmental milestones and personality functioning. To provide an explanatory framework for this link, it should be kept in mind that the concept of personality functioning was initially introduced to evaluate impairments and delays in the development of the adaptive intrapsychic system necessary to fulfill adult life tasks (Sharp & Wall, 2021). Moreover, many competencies that require mastery in adolescence can be framed within the domains of identity, self-direction, empathy, and intimacy: in short, personality functioning as conceptualized by Criterion A of the AMPD (Sharp, 2020). This conceptual overlap makes it theoretically plausible to expect the achievement of developmental milestones and personality functioning to be associated. Nevertheless, to our knowledge, only two works have empirically tested such a relation within the AMPD framework, thus measuring personality functioning as conceptualized in the DSM-5. One study pointed out that early adolescents’ difficulties in establishing supportive peer relationships predicted worse self-functioning in young adulthood (Vanwoerden et al., 2022); the other study, instead, found no association between discord in peer relationships and more severe personality functioning in 11–18-year-old participants (Skabeikyte-Norkiene et al., 2022). However, these studies are limited by, respectively, the consideration of only one domain of personality functioning (i.e., self-functioning) and the use of a cross-sectional design; in addition, both involved a non-clinical adolescent sample and focused only on one specific developmental task (i.e., establishing successful peer relationships). Given the contrasting results emerged from the available studies and the potential clinical relevance of investigating the interplay between attainment of developmental milestones and personality functioning in youth, it seems paramount to expand research in this direction.

## The Current Study

Although, as outlined above, there seem to be grounds to assume a close link between personality functioning, general psychopathology, and achievement of developmental milestones in youth, no study to date has considered all these variables in concert to examine their temporal dynamics. Therefore, the present research sought primarily to clarify the direction of the longitudinal associations between said constructs in a large mixed diagnostic sample of Dutch youth. To be more specific, the aim of the study was threefold: 1) to explore the temporal stability of each construct; 2) to investigate concurrent relations between the constructs; 3) to examine prospective relations, namely whether a) high levels of personality functioning impairment predicted high levels of general psychopathology 6 months later, or vice versa, or both ways; b) high levels of difficulties achieving developmental milestones predicted high levels of general psychopathology 6 months later, or vice versa, or both ways; c) high levels of difficulties achieving developmental milestones predicted high levels of personality functioning impairment 6 months later, or vice versa, or both ways. Generally speaking, it was expected that: each construct would be relatively stable over time (Hypothesis 1); high personality functioning impairment levels would be associated with high general psychopathology levels (and vice versa) at each time point (Hypothesis 2); high difficulties in the achievement of developmental milestones would be related to high personality functioning impairment (Hypothesis 3) and general psychopathology levels (Hypothesis 4) (and vice versa) at each time point. The final aim (i.e., the examination of prospective relations) was mainly exploratory since, to our knowledge, no previous study has addressed this research question yet; therefore, no specific hypothesis was formulated.

## Methods

### Participants and Procedure

The dataset used in this study was obtained by merging the data from two longitudinal projects on personality development in youth (Koster et al., 2022). In both studies, outpatient youth admitted to mental health care services in specialized institutions in The Netherlands were followed over time. In particular, data were collected on three occasions (referred to as T1, T2, and T3) within a year. Youth were referred by their general practitioner to these specialized mental health care institutes for different types of severe, often co-morbid, mental disorders, such as personality pathology and internalizing disorders (e.g., anxiety disorders, mood disorders, etc.). They underwent different types of treatment, primarily psychotherapeutic (e.g., cognitive behavioral therapy, schema-based therapy, family or group therapy, etc.), in some cases complemented with pharmacological treatment. Patients with an IQ below 85, schizophrenia spectrum disorders, acute suicidality or eating disorders were referred to other treatment programs and therefore not included in the longitudinal studies. The data used in the current study were collected with self-report measures as part of the routine outcome monitoring (ROM)-procedure, starting at intake at the institutions with a half-yearly follow-up. The information obtained from these measures was also used by clinicians to inform diagnostic assessment and treatment planning. Informed consent was obtained from patients (and caregivers when the patient was younger than 16 years of age), and patients agreed that the data could be used anonymously for research purposes (protocol numbers of the studies: FETC17- 092 and FETC17–090).

The sample obtained by merging the datasets of the two projects included 1226 White youth (72.5% self-identified women, age range: 12–26 years, *M* = 18.5 ± 2.83) who had participated in one or more waves of data collection (T1 and/or T2 and/or T3). However, given the longitudinal nature of the study, only patients who had also participated in at least a follow up (T2 or T3) were retained. Therefore, the final sample consisted of 525 youth (78.6% self-identified women, age range: 12–26 years, *M* = 18.8 ± 2.83), of whom 424 participated in T2 and 262 participated in T3. Patients who were excluded from the analytic sample (*n* = 701) did not differ from those included with respect to all study variables, except for personality functioning at T2 (*t* (319) = −2.11, *p* = 0.035); specifically, those included displayed higher levels of personality functioning impairment (*M* = 17.9, *SD* = 6.63) than those excluded (*M* = 14.9, *SD* = 5.67). In addition, when T1 measures were compared between youth who did or did not participate in T2, t-tests showed only a marginally significant difference in developmental milestones achievement (*t* (144) = −1.98, *p* = 0.049), with higher scores obtained by the former (*M* = 13.8, *SD* = 16.6 *vs. M* = 7.04, *SD* = 12.3). No differences were instead found when T2 measures were compared between youth who were or were not retained at T3. Therefore, it was assumed that the attrition was not systematic.

### Measures

#### Developmental Milestones List (DML; Laceulle et al., in progress)

This is a 21-item questionnaire including tasks and activities reflective of youth-specific developmental milestones. The items of this list ask, on a 7-point Likert scale, to what extent the participant experiences trouble in the achievement of youth-specific social (e.g., relationships with peers), personal (e.g., identity and autonomy), and professional (e.g., school/work) milestones (Spanjaard & Slot, 2015). Examples of items are, respectively, “Do you find it nice and important to make and have friends?”, “To what extent are you able to become independent?”, and “To what extent are you able to learn at school or training or do work well?”. These items combine into a total scale, which has been used in the present study. Cronbach’s alphas for the total scale ranged from 0.84 (T3) to 0.87 (T2) in the overall sample, from 0.84 (T1) to 0.87 (T2) in the female subsample, and from 0.87 (T1 and T3) to 0.91 (T2) in the male subsample.

#### Level of Personality Functioning Scale-Brief Form (LPFS-BF; Hutsebaut et al., 2016)

This is a 12-item self-report measure of personality functioning, which is divided into two subdomains of self-functioning and interpersonal functioning. A total score can also be calculated as a measure of global personality functioning level. Participants respond on a 4-point Likert scale ranging from *not at all true or often untrue* to *often true or completely true*. Examples of items are “I often do not know who I really am” and “My relationships and friendships never last long”. In the present study, the total score was used, which showed Cronbach’s alpha values ranging from 0.78 (T1) to 0.85 (T3) in the overall sample. Similar results were obtained when considering the female subsample (alphas from 0.79 (T1) to 0.86 (T3)) and the male subsample (alphas from 0.74 (T1) to 0.85 (T2)) separately.

#### General psychopathology

The Strengths and Difficulties Questionnaire (SDQ; Muris et al., 2003) and the Symptom Questionnaire-48 (SQ-48; Carlier et al., 2012) were administered to measure general psychopathology. Specifically, the SDQ is a 25-item self-report tool adopted to evaluate psychopathological symptoms in young people by means of four subscales: Emotional Problems, Conduct Problems, Hyperactivity, and Peer Problems. Participants are asked to indicate how much each statement describes them best using a 3-point Likert scale (0 = *Not true*, 2 = *Certainly true*). Examples of items are “I get very angry and often lose my temper”, “I am easily distracted, I find it difficult to concentrate”, “I am restless, I cannot stay still for long”, and “I am often unhappy, depressed, or tearful”. The scores on the abovementioned subscales can be summarized into a total score reflecting emotional and behavioral difficulties. More specifically, according to the Dutch language manual (Theunissen et al., 2019), a SDQ total score of 16 or greater (i.e., percentile score > 90) is considered high and could be indicative of underlying psychopathology. With specific reference to the present study, 73.7% of patients who completed the SDQ (*n* = 289) were above this cut-off. Cut-off scores for the subscales are as follows: Emotional Problems: cut-off = 6, 82% of this sample; Conduct Problems: cut-off = 4, 21.8% of this sample; Hyperactivity: cut-off = 7, 49.5% of this sample; Peer Problems: cut-off = 3, 58.8% of this sample. Then, as regards internal consistency, Cronbach’s alpha values for the total score ranged from 0.67 (T1) to 0.76 (T3) in the overall sample, from 0.64 (T1) to 0.76 (T3) in the female subsample, and from 0.61 (T1) to 0.74 (T3) in the male subsample.

The SQ-48 is a 48-item self-report questionnaire measuring psychological distress with 7 subdomains (depression, anxiety, somatic complaints, agoraphobia, aggression, cognitive problems, and social phobia), which can be combined into a total scale. All items are rated on a 5-point Likert scale ranging from *never* to *very often*. Examples of items are “I had trouble with controlling my anger”, “I could not concentrate well”, “I felt restless”, and “I felt down or depressed”. Cut-off scores for the SQ-48 subscales (I. Carlier et al., 2012) and percentages of patients (*n*_total_ = 218) who scored above them are as follows: Depression: cut-off = 4, 95%; Anxiety: cut-off = 6.5, 90.4%; Somatic complaints: cut-off = 1.5, 89.9%; Agoraphobia: cut-off = 0.5, 79.8%; Aggression: cut-off = 1.5; 80.3%; Cognitive problems: cut-off = 7.5, 85.3%; Social phobia: cut-off = 3.5, 87.6%. The SQ-48 was shown to have excellent psychometric properties (I. Carlier et al., 2012; I. V. E. Carlier et al., 2017). As for the present study, Cronbach’s alpha values for the total score ranged from 0.94 (T1) to 0.96 (T2 and T3) in both the overall sample and the separate subsamples of female and male individuals.

Patients who completed the SDQ did not complete the SQ-48, and vice versa. This distinction was due to the fact that the questionnaires have been originally developed for different populations, i.e., the SDQ for children and adolescents younger than 18 years of age, while the SQ-48 for people aged 18 years or older. However, both measures encompass aspects of internalizing and externalizing pathology, with relatively more items referring to internalizing pathology. In addition, the total scores of the SDQ and SQ-48 assess the same overarching construct, that is psychological distress or general psychopathology. Research with other psychological distress questionnaires (Patient Health Questionnaire-4, Kessler-10/Kessler-6, Distress Questionnaire-5, Mental Health Inventory-5, Hopkins Symptom Checklist-25, Self-Report Questionnaire-20, and Distress Thermometer) pointed out strong correlations (*r* > 0.80) between the total scores of these measures, with a single (first) factor explaining more than 70% of the variance in most cases (Batterham et al., 2018). Moreover, for example, the correlation between the total scores of the Child Behavior Check List 6–18 and the SDQ was found to be high (*r* > 0.80), also showing that “an overwhelming majority of total score variance in all domains and samples is attributable to the general factor” (Mansolf et al., 2022; p.238). Therefore, different measures of psychological distress in adult and youth samples tend to correlate strongly and seem to capture the same (general distress) variance. While this assumption cannot be empirically tested in the present sample, it is reasonable to infer, based on existing literature, that the SDQ and SQ-48 total scores refer to comparable psychological distress variance. Therefore, for the purpose of the analyses, a single scale called “General Psychopathology” (GP) was calculated by combing the standardized total scores of the SDQ and SQ-48. In fact, fitting two separate models for adolescents (SDQ) and emerging adults (SQ-48) would have resulted in a loss of statistical power since the sample size for each model would have been considerably reduced.

### Data Analysis

#### Preliminary analyses

The data were first checked for univariate normality using the Shapiro-Wilk test. The ranges of skewness and kurtosis were also evaluated and considered acceptable if between ± 0.5 and ± 3, respectively. Pearson’s *r* correlations were then calculated at each time point for age, the GP scale, and the standardized total scores of the DML and LPFS-BF (i.e., the measures used in the subsequent model). In this regard, *r* values were interpreted as a measure of effect size on the basis of Cohen (1988)’s criteria: 0.10 ≤ *r* < 0.30 indicates a small effect, 0.30 ≤ *r* < 0.50 a moderate effect, and *r* ≥ 0.50 a large effect. Subsequently, Little’s MCAR test was run to exclude that missing data patterns violated the assumption of non-random distribution. If the test is not statistically significant (i.e., missingness is completely at random), model parameters can be estimated considering all available cases, with missing values imputed using Full Information Maximum Likelihood (FIML).

#### Main analyses

A Cross-Lagged Panel Model (CLPM; Campbell & Kenny, 1999; Rogosa, 1988) with Maximum Likelihood (ML) as estimator was employed to analyze the longitudinal relations between developmental milestones (i.e., standardized DML total score), personality functioning (i.e., standardized LPFS-BF total score), and general psychopathology (i.e., GP scale). Age and gender were added as covariates for each measure at T1 due to the wide age range and the predominance of women in the sample. The choice of a CLPM over methods focusing on within-person change (e.g., Random Intercept-Cross-Lagged Panel Model, RI-CLPM) was based on both theoretical and methodological reasons. First, the present study was aimed at specifically detecting prospective between-person processes (namely, whether youth high on a particular risk factor will present a worse outcome than youth low on that risk factor) and, according to the literature, this objective can be successfully addressed by using a CLPM approach. Indeed, models like RI-CLPMs assess prospective effects of within-person fluctuations around the trait level in a construct, thus not allowing one to answer questions about the effects of between-person differences (Asendorpf, 2021; Orth et al., 2021). To give an example in the context of this study, through a CLPM it is possible to test whether youth with high difficulties in achieving developmental milestones (relative to others) will experience a subsequent increase in general psychopathology compared to youth with low difficulties in achieving developmental milestones. In contrast, cross-lagged effects in RI-CLPMs would indicate whether youth with higher difficulties achieving developmental milestones than usual at a specific time point will experience an increase in general psychopathology at the next time point. From a methodological perspective, we refrained from using a RI-CLPM approach because this model requires large sample sizes (i.e., >1000) to accurately detect even moderate effect sizes (Masselink et al., 2018), so the current study’s sample was not large enough. Moreover, data were collected on three measurement occasions, while it has been recognized that that more than three data points are needed to properly control for the influence of measurement error in RI-CLPMs (Park et al., 2023; Usami et al., 2019; Lüdtke & Robitzsch, 2021). However, to empirically test all these methodological issues, an RI-CLPM was also fitted to the data: the model did not converge (the output can be found at the Open Science Framework (OSF) page linked at the end of the paragraph), thus supporting the unsuitability of an RI-CLPM approach for the research design of the present study.

To achieve the most parsimonious CLPM, an initial fully free CLPM was compared with CLPMs in which paths were constrained to be equal over time, one-by-one in the order of stability, cross-lagged, and concurrent effects (Kim et al., 2022; Orth et al., 2021). In other words, a CLPM was first estimated allowing all effects to vary across waves, so they were free to differ at each of the three time points. Then, this baseline model was compared with (a) a model in which autoregressive paths from T1 to T2 were equal to the same associations from T2 to T3 (e.g., the path from DML at T1 to DML at T2 was equal to the path from DML at T2 to DML at T3); (b) a model in which autoregressive and cross-lagged effects from T1 to T2 were equal to the corresponding paths from T2 to T3 (e.g., the path from DML at T1 to DML at T2 was equal to the path from DML at T2 to DML at T3, as well as the path from DML at T1 to LPFS-BF at T2 was equal to the path from DML at T2 to LPFS-BF at T3); (c) a fully constrained model in which, together with autoregressive and cross-lagged paths (see example above), also concurrent paths were set to be equal across waves (e.g., the association between DML and LPFS-BF at T1 was equal to the association between DML and LPFS-BF at T2 and T3). The fit of each model was evaluated considering the Comparative Fit Index (CFI), Root Mean Square Error of Approximation (RMSEA), and Standardized Root Mean Square Residual (SRMR). CFI values greater than 0.90 and 0.95, and RMSEA and SRMR values below 0.080 and 0.050 were considered indicative of, respectively, acceptable and good fit (Bentler & Bonett, 1980; Hu & Bentler, 1999). In particular, after constraining each path, the model fit was compared following the recommendations by Chen (2007), according to which invariance over time can be considered supported when ΔCFI < 0.010, ΔRMSEA < 0.015, and ΔSRMR < 0.030. If the model fit became notably worse after constraining a path, that constraint was removed.

CLPM analyses were conducted using the *lavaan* R package (Rosseel, 2012). The R syntax and output are openly available via https://osf.io/uz3gy/?view_only=e19779dffe29492f8cb8b6f028429c9b.

## Results

### Descriptive Results

Descriptive statistics for each of the administered questionnaire are reported in Table 1. The Shapiro-Wilk test was statistically significant for DML and SQ-48 at T1, and LPFS-BF at T3; nevertheless, the skewness and kurtosis values indicated that the data were quite symmetrical. The same applies to the GP scale: the Shapiro-Wilk test was statistically significant at T1 and T2 (*W* = 0.99), but the distribution was relatively symmetrical, as evident from the skewness (T1: −0.24; T2: −0.09; T3: −0.06) and kurtosis (T1: −0.23; T2: −0.50; T3: −0.54) values.

**Table 1:** Descriptive Statistics of the Administered Questionnaires

	*N*	*M* (*SD*)	Skewness	Kurtosis	*W*
DML T1	146	12.5 (16.1)	0.19	−0.56	0.98*
DML T2	153	15.5 (15.8)	0.09	−0.28	0.99
DML T3	101	18.6 (14.3)	0.42	−0.15	0.98
LPFS-BF T1	350	18.1 (6.2)	−0.07	−0.24	0.99
LPFS-BF T2	298	17.9 (6.6)	−0.08	0.13	0.99
LPFS-BF T3	207	16.4 (7.1)	−0.16	−0.58	0.99*
SDQ T1	289	18.6 (4.76)	−0.23	−0.05	0.99
SDQ T2	240	17 (5.09)	−0.08	−0.42	0.99
SDQ T3	150	16.8 (5.45)	−0.03	−0.33	0.99
SQ T1	218	71.1 (25.6)	−0.27	−0.39	0.99*
SQ T2	166	67.2 (28.7)	−0.09	−0.54	0.99
SQ T3	106	64.7 (29.3)	−0.09	−0.72	0.99

*DML* Developmental Milestones List, *LPFS-BF* Level of Personality Functioning Scale-Brief Form, *SDQ* Strengths and Difficulties Questionnaire, *SQ-48* Symptom Questionnaire-48

*p < 0.05

Table 2 shows the bivariate correlations between the continuous variables used to fit the CLPM (i.e., age, the GP scale, and the standardized scores of the LPFS-BF and DML). The correlations within time (i.e., at each time point) between the DML and both the LPFS-BF and GP scale were large and negative, while those between the LPFS-BF and GP scale were large and positive. The lowest *r* values were between different measures at different time points. Age was moderately associated with DML at T2, while correlations with the other constructs showed a small or non-significant effect.

**Table 2:** Pearson’s *r* Correlations Between the Continuous Variables Included in the CLPM

	1.	2.	3.	4.	5.	6.	7.	8.	9.
1. DML T1	–								
2. DML T2	0.68*	–							
3. DML T3	0.64*	0.72*	–						
4. LPFS-BF T1	−0.51*	−0.16	−0.14	–					
5. LPFS-BF T2	−0.51*	−0.46*	−0.53*	0.70*	–				
6. LPFS-BF T3	−0.33*	−0.36*	−0.66*	0.43*	0.80*	–			
7. GP T1	−0.67*	−0.38*	−0.26*	0.70*	0.57*	0.26*	–		
8. GP T2	−0.40*	−0.56*	−0.39*	0.42*	0.75*	0.53*	0.59*	–	
9. GP T3	−0.39*	−0.43*	−0.60*	0.23*	0.69*	0.75*	0.31*	0.74*	–
10. Age	0.07	0.30*	0.13	0.24*	0.06	0.003	−0.01	−0.13*	−0.24*

*DML* standardized total score of the Developmental Milestones List, *LPFS-BF* standardized total score of the Level of Personality Functioning Scale-Brief Form, *GP* total score of the General Psychopathology scale

*p < 0.05

Finally, pertaining to missing data analysis, Little’s MCAR test was not significant (χ^2^ = 205.75, df = 184, *p* = 0.13), suggesting that the risk of attrition bias was negligible. Therefore, FIML estimation was used in the following analyses to account for missing values.

### Main Analyses

The initial CLPM (i.e., Model 1), testing the unique associations between achievement of developmental milestones, personality functioning, and general psychopathology while controlling for gender and age, provided an excellent fit to the data (Table 3). Then, the autoregressive, cross-lagged, and concurrent paths were constrained to be time invariant one-by-one (Models 2 to 5). As can be seen in Table 3, the fit of the models where the autoregressive and cross-lagged effects were constrained to be equal over time (Models 2 and 3) remained acceptable. However, the fit became notably worse when adding constraints to the concurrent paths (i.e., concurrent associations at T1 were set to be equal to the corresponding associations at T2 and T3; Model 4). This outcome suggests that the bivariate associations between constructs (i.e., between developmental milestones and personality functioning, personality functioning and general psychopathology, and developmental milestones and general psychopathology) are not equal across all the three time points. From an interpretative perspective, Model 4 fails to capture the longitudinal changes occurring during different phases of treatment, which can be reflected in how the variables relate to each other. In light of these considerations, constraints to the concurrent associations at T1 were removed (i.e., concurrent paths at T1 were allowed to differ from the same associations at T2 and T3, which instead were held to be equal; Model 5). This modification improved the fit and made it not markedly different from the fit of the model with stability and cross-lagged paths constrained. This means that the relations between the constructs at T1 are not equal to these same relations at T2 and T3; in other words, concurrent relation patterns remain consistent and equal between T2 and T3, but different from T1. To interpret this finding, it should be considered that at T1 patients were at the beginning of treatment; therefore, it is plausible that therapeutic changes are more strongly visible at this time point (i.e., early stage of clinical treatment) and then tend to stabilize and consolidate later (T2 and T3) (Owen et al., 2015). In the context of the present study, these different trajectories of change could result in different patterns of relations among constructs, depending on the treatment stage (i.e., early *vs*. after 6 and 12 months). Thus, to sum up, Model 5, in which concurrent associations at T1 were free to vary and the other paths were all constrained to be equal over time, was chosen as the parsimonious CLPM. The analyses supported the longitudinal invariance of the autoregressive and cross-lagged paths, and the partial invariance of the concurrent correlations.

**Table 3:** Fit Comparisons of CLPMs With and Without Constraints Over Time

Model	CFI	TLI	RMSEA	(90% CI)	SRMR	Comparison	Constrained*
Model 1: Unconstrained model	0.979	0.945	0.052	(0.034–0.071)	0.029		
Model 2: Stability paths constrained	0.966	0.932	0.058	(0.043–0.074)	0.038	vs. Model 1	Accepted
Model 3: Model 2 + cross-lagged paths constrained	0.968	0.947	0.051	(0.037–0.066)	0.040	vs. Model 2	Accepted
Model 4: Model 3 + concurrent paths constrained	0.936	0.911	0.067	(0.054–0.080)	0.101	vs. Model 3	Rejected
Model 5: Model 4 without concurrent paths at T1 constrained	0.965	0.948	0.051	(0.037–0.065)	0.039	vs. Model 3	Accepted

Model 2 vs. Model 1: ΔCFI = 0.013, ΔRMSEA = 0.006, ΔSRMR = 0.009; Model 3 vs. Model 2: ΔCFI = 0.002, ΔRMSEA = 0.007, ΔSRMR = 0.002; Model 4 vs. Model 3: ΔCFI = 0.032, ΔRMSEA = 0.015, ΔSRMR = 0.061; Model 5 vs. Model 3: ΔCFI = 0.002, ΔRMSEA = 0, ΔSRMR = 0.001

*Only accepted paths were constrained; rejected paths were allowed to vary over time

**Fig. 1:**
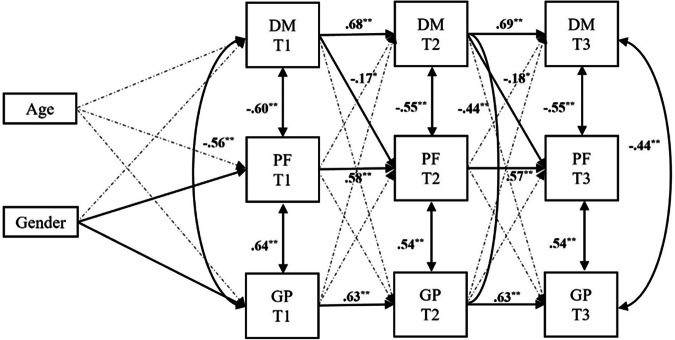
Simplified cross-lagged panel model examining the longitudinal associations between achievement of developmental milestones (DM), personality functioning (PF), and general psychopathology (GP) controlling for age and gender. *Note*. Dashed paths indicate non-significant estimates, while bold paths represent significant effects. The values displayed are standardized coefficients. *p < 0.010, **p < 0.001

As shown in Fig. 1, all the autoregressive paths were positive and significant, indicating moderate stability over time. Similarly, the concurrent relations were all significant with large effects, suggesting associations between the constructs. To be specific, the LPFS-BF and GP scale were positively associated within time point, pinpointing that people who scored high in personality functioning impairment also scored high in general psychopathology (and vice versa). Instead, the DML was negatively linked to both the GP scale and LPFS-BF at each time point, meaning that youth with high difficulties in achieving normative developmental milestones also presented elevated levels of general psychopathology and impairments in personality functioning (and vice versa). With regard to cross-lagged paths, significant and negative effects were found from DML at T1 and T2 to, respectively, LPFS-BF at T2 and T3. Thus, patients with high difficulties in achieving developmental milestones were more likely to present a worsening in personality functioning 6 months later compared to patients with low difficulties in achieving developmental milestones. No evidence of the opposite association emerged, as well as of other cross-lagged effects. Finally, gender was found to be associated with the LPFS-BF (β = 0.32, *p* = 0.008) and GP scale (β = 0.44, *p* < 0.001) at T1, while no effect of age was detected. Specifically, female patients were found to be more likely to obtain higher scores on these measures compared to male patients.

## Discussion

While there is theoretical evidence to support the existence of a link between personality functioning, general psychopathology, and achievement of youth developmental milestones, inadequate attention has been directed to investigating these variables together to clarify their mutual influences. Nevertheless, delving into this topic is clinically relevant given the high prevalence of mental disorders in adolescence and emerging adulthood (Blakemore, 2019). In particular, such an exploration can offer valuable insights into the priority of change between the above constructs, thus enabling the identification of prospective targets for prevention and treatment interventions. Building on these premises, the current study involved a large outpatient sample of youth to unravel the longitudinal relations between personality functioning, general psychopathology, and achievement of developmental milestones.

First, the autoregressive paths revealed a moderate stability over time of the variables considered, indicating that the level of each construct was not absolutely stable, but fluctuated somewhat from T1 to T2 and from T2 to T3. This is consistent with Hypothesis 1 and with what might be expected in a sample of vulnerable youth in a clinical context. Indeed, on the one hand, these fluctuations could be explained in light of the many social and physical challenges that characterize adolescence and emerging adulthood (e.g., Elder Jr. & Shanahan, 2006). On the other hand, youth were undergoing some type of treatment, which might have contributed to the fluctuations over time in the level of the constructs. Overall, these results would suggest that, although the level of the constructs seems to be relatively stable over 6 months, it can also fluctuate consistently with the rapid and continuous maturational changes that make this life stage malleable per se (Steinberg et al., 2015) and/or with the presence of ongoing treatment. Notably, this highlights that personality functioning, general psychopathology, and achievement of developmental milestones are constructs amenable to change in youth, thus supporting the importance of studying their trajectories and including them as targets of interventions. In particular, personality dysfunction, psychopathological symptoms, and difficulties achieving developmental tasks appear to be modifiable risk factors; hence, given the high vulnerability of adolescence and emerging adulthood, preventive endeavors addressing these factors could prove effective in protecting youth from subsequent maladaptive outcomes. Nevertheless, it should also be considered that the time lag between measurement waves was relatively short, so it cannot be excluded that stability decreases by considering a wider time interval. Replicating these findings by conducting more extended longitudinal studies is thereby highly recommended.

The hypotheses were also confirmed as regards the concurrent relations (Hypotheses 2 to 4), which were all significant with large effects. To be specific, personality dysfunction and general psychopathology were positively associated at each time point, indicating that youth with severe impairment in personality functioning also had elevated levels of general psychopathology (and vice versa). These data are particularly intriguing, as they contribute to widening the limited literature on the link between personality functioning and mental disorders in adolescence and emerging adulthood. Specifically, they suggest that impaired personality functioning and general psychopathology can co-occur in youth, aligning with the studies that have underscored the clinical utility of thoroughly evaluating personality functioning in relation to broader spectra of psychopathology (Doubková et al., 2022; Sleep et al., 2019). Moreover, youth with impairments in personality functioning may also present high levels of general psychopathology, which should be assessed and targeted in interventions. Therefore, the evaluation of both personality functioning and general psychopathology seems essential for a comprehensive diagnostic process, as well as for the formulation of an effective treatment plan for young patients. In particular, therapeutic strategies designed to enhance personality functioning may be a useful addendum to conventional treatments for psychopathology in young patients, as well as interventions addressing general psychopathological symptoms may promote improvements in personality functioning.

Subsequently, negative concurrent associations were found between the achievement of developmental milestones and general psychopathology. This replicates cross-sectional findings (Hirota et al., 2022; Ogilvie et al., 2019), specifically displaying that youth who had difficulties achieving age-typical developmental tasks were also high in general psychopathology levels (and vice versa). Therefore, it could be that failure to complete developmental tasks induce general psychopathology; at the same time, it is also possible that the presence of psychopathology interferes with the successful mastery of developmental milestones (Pinquart & Pfeiffer, 2020). A viable theoretical interpretation of this result is offered by the Common Cause Models, which posit that problems with the achievement of phase-specific developmental tasks and psychopathology may share the same underpinning mechanisms manifesting in different ways (Masten et al., 2015). Future investigations should thereby deepen this topic by also considering the variables that might be involved in the relation between general psychopathology and attainment of developmental tasks (e.g., temperament, parental bonding, personality traits, etc.). Clinically speaking, this finding would suggest that treatment programs promoting age-adequate functioning in young patients may be helpful in simultaneously reducing general psychopathology, thus facilitating improvements and their maintenance after treatment. Furthermore, it should be kept in mind that young people who struggle to attain developmental tasks may also experience psychological distress; therefore, interventions aimed at fostering a successful resolution of stage-salient developmental milestones should also target co-occurrent general psychopathology/distress.

Negative concurrent associations also emerged between the achievement of developmental milestones and personality dysfunction, pinpointing that youth facing difficulties attaining normative developmental tasks were also highly impaired in personality functioning (and vice versa). However, the key finding in this regard stems from the cross-lagged paths, which showed that high difficulties in the achievement of developmental milestones predicted a worsening in personality functioning 6 months later, while the opposite effect was not supported. This evidence is interesting, as it highlights that the achievement of youth-specific developmental milestones precedes personality functioning, thus expanding on previous research assuming the existence of a relation between these constructs (Sharp, 2020; Vanwoerden et al., 2022). A possible explanation is based on Sharp (2020)’s observations, according to which many of the competences developed in adolescence relate to the domains included in the concept of personality functioning, namely identity, self-direction, empathy, and intimacy. Consequently, problems in successfully mastering age-typical developmental tasks (e.g., identity formation, gaining autonomy, and establishing satisfying interpersonal relationships) could be a factor that contributes to undermining the foundation for the subsequent development of adaptive personality functioning. Furthermore, referring to the models outlined by Masten et al. (2015), the present findings align with the Failure Models, suggesting that challenges in completing developmental tasks contribute to subsequent psychological difficulties, specifically in terms of impaired personality functioning. This would imply that the opposite models (i.e., Psychopathology undermines competence) do not hold, at least in the context of the relation between developmental milestones and personality functioning. However, drawing definitive conclusions on the other models based on the current data is not feasible. For example, the Common Cause Models could also hold validity, as it seems plausible that difficulties in meeting developmental milestones and impaired personality functioning may arise from shared processes (e.g., invalidating contexts experienced by youth) that influence the complex dynamic underlying adaptive development. Therefore, further research is imperative to afford a more nuanced understanding of this matter. In particular, a systematic comparison of different models becomes essential to elucidate developmental mechanisms and, ultimately, guide future intervention efforts. To this end, subsequent studies could consider other variables (such as contextual or regulation variables) to investigate their role in the (longitudinal) relation between developmental tasks achievement and personality functioning.

From a clinical perspective, the predictive role of developmental milestones achievement on personality functioning suggests that considering developmental tasks as targets of treatment programs could be a promising avenue to promote better personality functioning and psychological well-being in youth. Therefore, mental health interventions should shift the focus from merely reducing clinical symptomatology (i.e., a pathology perspective) to fostering the attainment of developmental competencies to promote self- and interpersonal functioning (i.e., a developmental perspective) (Holmbeck et al., 2006; Ialongo et al., 2015). The importance of this intervention strategy is underscored by recent findings showing that personality functioning (Criterion A) strongly predicted disability and symptom severity one year later (Weekers et al., 2023). The current data also suggest that specific attention should be paid to young people with difficulties achieving normative developmental milestones, as they may be vulnerable to later impairment in personality functioning and, more broadly, psychological maladjustment; hence the relevance of implementing early and targeted preventive efforts aimed at these individuals. All these purposes can be addressed by assessing developmental milestones achievement in a variety of contexts (e.g., clinical practice, schools, etc.). The introduction of developmentally sensitive screeners, for example, would allow for the monitoring of developmental progression, identification of at-risk youth, and early-intervention for maladaptive trajectories. Furthermore, given that the negotiation and mastery of developmental tasks occur amongst several figures and within several processes and domains, it is imperative to implement multimodal and multidisciplinary interventions to mitigate the risk of unfavorable long-term outcomes. Thus, standard treatment and prevention programs for personality dysfunction in adolescence and emerging adulthood may benefit from the inclusion of modules aimed at equipping youth with more functional self-management, school-based, and social skills and promoting positive relationships with peers and parents (e.g., life and interpersonal skills training, psychoeducation, interaction-based strategies, etc.). In light of this, studies that develop and test the effectiveness of interventions designed to promote the attainment of stage-salient developmental milestones to specifically treat or prevent maladaptive personality functioning in young people are encouraged.

Finally, a result worthy of attention is the link between gender and both personality functioning impairment and general psychopathology, with female patients being more likely to exhibit high levels of these constructs compared to male patients. This finding can be explained on the basis of the following considerations. First, men and women seem to differ in how they cope with stressors. Specifically, research has shown that women would perceive the same stressor as more intense compared to men (Jose & Ratcliffe, 2004). Moreover, men tend to use avoidance or distraction as coping strategies (Seiffge-Krenke, 2013), while women’s responses are often characterized by rumination and a tendency to seek social support (Eschenbeck et al., 2007). Additionally, societal gender norms and stereotypes about masculinity (e.g., “men must be strong”), coupled with the stigma surrounding mental health (e.g., “people with mental disorders are weak”), may cause men to feel shame and struggle to admit and express psychological problems (Yoon et al., 2023; Mannarini et al., 2023). Overall, these factors may play a role in the self-reported lower rates of psychopathological symptoms and personality functioning difficulties in male patients. However, to our knowledge, a systematic understanding of if and how gender influences personality functioning and general psychopathology in youth is still lacking; thus, this study may serve as a useful starting point for future research.

All in all, the present findings should be interpreted in the context of the study’s limitations. First, the sample included only Dutch outpatients and lacked a control group consisting of healthy youth, thus limiting the generalizability of the findings to the whole adolescent and emerging adult population, as well as to adolescent and emerging adult inpatients. Future studies should be conducted in different cultural and clinical contexts, also considering non-clinical youth. In addition, the fact that the patients were diagnosed with a wide array of psychological disorders may have affected the results. Again, in terms of sample characteristics, the age range was wide and encompassed different stages of adolescence and young adulthood; additional studies should thereby be conducted to investigate the longitudinal relations between the constructs by specifically distinguishing between different developmental phases. Moreover, the reliance on self-reported measures has potentially introduced common-method variance; future investigations could benefit from using multiple method designs (e.g., observational) and multi-informant tools to enhance the robustness of the findings. It is also worth noticing that attrition rates over time were high, and that only a subset of patients participated at all the three time points, possibly due to the clinical nature of the sample. An additional limitation is that Cronbach’s alpha value of the SDQ at T1 was not excessively high, although it was within the acceptable range according to the latest guidelines (Taber, 2018). Finally, the use of CLPMs requires caution in interpreting causal effects (Lucas, 2023); therefore, future research should consider replicating this study by assessing the constructs across multiple time points (e.g., experience sampling methods) and then adopting an RI-CLPM analytic approach. This design would enable the evaluation of individual temporal fluctuations as well (Hamaker et al., 2015), thus broadening the understanding of differences within and between persons and contributing to the ongoing scientific discussion over the applicability of both CLPM and RI-CLPM when testing causal hypotheses (Asendorpf, 2021; Lüdtke & Robitzsch, 2021). Despite such limitations, the present study is characterized by notable strengths, the main ones being the involvement of a large sample of referred youth, the use of a longitudinal design, the adoption of a developmental perspective, and the consideration of variables never investigated together before. These aspects make the results innovative and helpful, as they suggest valuable avenues for future research and clinical interventions in the realm of youth psychopathology.

## Conclusion

Although personality functioning, general psychopathology, and achievement of developmental milestones are both unique and related critical domains in the field of youth mental health, little empirical evidence is available on this topic. Specifically, to our knowledge, no research to date has considered these constructs jointly or examined the temporal associations between them. This longitudinal research contributed to fill these gaps by providing empirical support to the interplay between personality functioning, general psychopathology, and achievement of developmental milestones in clinical youth. In particular, the achievement of developmental tasks was found to predict personality functioning 6 months later. This crucial finding emphasizes the pivotal role played by the attainment of phase-specific developmental tasks in the establishment of personality functioning and thus the relevance of adopting a developmentally sensitive approach to properly frame and understand topics related to young people’s mental health. In terms of clinical practice, the successful mastery of developmental milestones would seem to be a protective factor against the onset of maladaptive personality functioning. Therefore, efforts directed at increasing age-adequate functioning and addressing barriers to completing developmental tasks could be effective ways to prevent later personality functioning impairments or to counteract/ameliorate its negative sequelae. Finally, inherent in the adoption of a developmental viewpoint is the need for early recognition of vulnerable youth; therefore, those who struggle to achieve stage-salient developmental tasks should be timely detected, in order to implement tailored and more intensive interventions, prevent negative outcomes, and promote access to adaptive development pathways.
